# The Road from Pathological Narcissism to Suicidality in Adolescence: An Empirical Study

**DOI:** 10.3390/ijerph18189761

**Published:** 2021-09-16

**Authors:** Riccardo Williams, Maria Pia Casini, Marta Moselli, Camilla Frattini, Elsa Ronningstam

**Affiliations:** 1 Department of Dynamic, Clinical Psychology and Health Studies, “Sapienza”—University of Rome, 00185 Rome, Italy; marta.moselli@uniroma1.it (M.M.); camilla.frattini@uniroma1.it (C.F.); 2 Child Neuropsychiatry Unit, Department of Neuroscience, I.R.C.C.S. Children Hospital Bambino Gesù, 27100 Rome, Italy; m.casini@policlinicoumberto1.it; 3 Child and Adolescent Neurology and Psychiatry Unit, Department of Human Neuroscience, “Sapienza”—University of Rome, 00185 Rome, Italy; 4 Harvard Medical School, McLean Hospital, Belmont, MA 02478, USA; ronningstam@email.com

**Keywords:** adolescence, suicidality, narcissism, motivation, personality

## Abstract

Background: Clinical and empirical research evidenced a complex link between pathological narcissism and the suicidal process in adulthood. Given the relevance of suicidality and the peculiar narcissistic vicissitudes of adolescence, the proposed research investigated the relationship between pathological narcissism analyzed from the multi-dimensional perspective of the Diagnostic Interview for Narcissism (DIN) and suicidal ideation conducted in a sample of Italian Adolescents. Methods: One hundred and three Italian male and female adolescents between 12 and 18 were administered the DIN, SCIDII, CSSRS, and Kiddie-SADS with six months follow-up. Results: The correlation, *t*-test, multiple regression analyses evidenced the association of narcissistic affective states and mood with both suicidal ideation and lethality of conduct. The increase in the dimension of grandiosity is associated with the passage to potentially highly lethal suicidal gestures. Conclusions: Suicidal ideation and conduct seem to serve the function of restoring a sense of control and self-esteem in narcissistic individuals experiencing a state of affective dysregulation. Narcissistic pathological functioning seems to play an important role in the adolescent suicidal process, quite like adulthood. Assessing an adolescent’s narcissistic functioning may provide useful clinical information in understanding and managing the suicidal risk in this phase of life.

## 1. Introduction

### Pathological Narcissism and the Suicidal Process

According to the latest available report from the World Health Organization (WHO) [1], suicide is the third leading cause of death in young people between the ages of 15 and 19, for both sexes, and the maximum peak incidence of suicidal ideation and behaviors is indeed observed during adolescence [2]. With reference to the consequences of the COVID-19 epidemic, recent studies indicate that the rates of suicidal ideation and suicidal behaviors between the ages of 11 and 19 were significantly higher for some months of 2020 compared to 2019 [3].

Empirical studies show that any risk factor considered on its own seems to account only for a small percentage of suicides. Current orientations prefer to identify configurations of different risk factors interacting with each other in a sequential process that begins before the actual suicidal behavior takes place and even before the suicidal ideation becomes conscious [4]. This process starts with a motivational phase in which the early idea of self-death is gradually transformed into suicidal ideation possibly leading to the real suicidal intention and planning. Many subjects report having nurtured suicidal ideation and even intent without having resorted to suicidal behavior—the staging model of the suicidal process thus describes a volitional phase subsequent to the motivational one [5]. The contribution of personality pathology could be understood with respect to its role in facilitating the passages through each stage of the suicidal process. The presence of specific personality disorders may in fact represent an activating factor [6,7,8] for stressful events [9,10,11,12,13,14], as well as a facilitating factor for the passage from the volitional phase to the motivational one [15,16]. Both in adulthood and adolescence [17], Cluster B personality disorders are the most significant predictors of suicide [8] with a prominent role being assigned to the borderline personality disorder (BPD) and its clinical traits [18,19,20,21,22,23,24]. 

Both clinical and research evidence indicates a role for narcissistic personality for both the recurrence of suicidal ideation and lethality of suicidal attempts in adulthood [25,26,27,28] and adolescence [29]. Regrettably, only marginal attention has been paid to the relationship between pathological narcissism and suicidality in adolescence [17], despite robust indications for the impact and diagnosticability of such pathological organizations of personality in this phase of development [29,30,31]. It should be considered that the impact of narcissistic functioning for adjustment in adolescence cannot be overestimated. The cognitive [32], social [33], and bodily and motivational [34] changes occurring in this phase determine spasmodic and agitated attention toward the self and relatedness, where the former resources for the regulation of self-esteem are not any longer guaranteed and new ones are still under construction [35]. Under specific internal or external pressures, this condition of enhanced narcissism may shift toward a state of narcissistic vulnerability and altered narcissistic functioning, also in adolescents who do not meet the criteria for a narcissistic personality disorder (NPD) diagnosis [36].

In this paper, the potential role of narcissistic pathological functioning for suicidality has been investigated in a sample of Italian adolescents in order to enhance the understanding and management of this severe clinical challenge in this phase of life.

In general, empirical studies have individuated some specific characteristics of the connection between narcissistic pathological functioning and suicidality, with some issues that still remain controversial and others that need further investigation. The relatively modest extant research seems to suggest that NPD is associated with repeated attempts and greater risk of death and of highly lethal suicide attempts, but is either not associated or moderately protective against lower lethality attempts [37]. At an empirical level, some studies have reported the contribution of narcissistic pathology for both the recurrence of suicidal ideation and lethality of suicidal attempts in adulthood [25,26,38], also in the absence of other risk factors including other relevant psychopathological conditions such as mood disorders [16]. It has also been shown that the narcissistic pathology may increase suicidal risk in combination with other factors such as psychopathological conditions or stressful life events [25]. Suicidal ideation is reported to occur frequently in narcissistic individuals, often without hesitating in actual anti-conservative acts [39]. Current research seems to suggest that NPD is associated with less frequent but more lethal attempts, but it is either not associated or moderately protective against lower lethality attempts [25,37].

Some of these more controversial issues have been dealt with from a more theoretical and clinical perspective. From this perspective, the emphasis is posed upon the function that the diverse steps of the suicidal process may acquire for the regulation of emotions and self-esteem in narcissistic individuals [40].

In particular, suicidal ideation is interpreted as “a first line strategy”, that comes into play to help narcissistic individuals recover their self-esteem by establishing emotional control, a sense of agency, and personal mastery [25]. When the feelings of anxiety, fear, shame, sadness, rage, and envy escalate, or when external events overwhelm the protective function of suicidal ideation, the narcissistic vulnerability becomes unbearable and can only be dealt with through actual suicidal planning and attempt [28,41]. This theoretically and clinically informed model of narcissistic suicidality has, so far, not undergone direct empirical scrutiny. Authors supporting this perspective emphasize the necessity for empirical research to study the characteristics of pathological narcissism well beyond the categorical approach provided by the Diagnostic and Statistical Manual of Mental Disorders (DSM) criteria for NPD [42,43].

The general aim of this work is to therefore investigate the relationship between narcissistic pathological functioning, observed from a multi-dimensional perspective, and suicidality in adolescence. Three main research objectives have been established: (1) to verify which aspect of pathological narcissism as assessed through the diagnostic interview for narcissism (DIN) are associated (correlate with) suicidal ideation (severity and intensity) as assessed through the Columbia Suicide Severity Rating Scale (CSSRS); (2) to verify which aspect of pathological narcissism as assessed through the DIN distinguish pure ideators from attempters at a longitudinal level; (3) to verify whether and which aspects of pathological narcissism contribute more to the potential lethality of the suicidal conducts.

## 2. Materials and Methods

### 2.1. Assessment

Mapping pathological narcissism as a multi-dimensional construct: the Diagnostic Interview for Narcissism.

The Diagnostic Interview for Narcissism [44] is a semi-structured clinician report interview. The DIN is the first instrument that has been developed from and applied to clinically diagnosed narcissistic patients. The aim of this instrument is the examination of narcissism as a pathological dimension of personality (i.e., as a trait), but it is suitable for diagnosing NPD. The content of the interview is divided into five sections: (I) Grandiosity, (II) Interpersonal relations, (III) Reactiveness, (IV) Affects and mood states, and (V) Social and moral adaptation. Each of the five sections is evaluated separately and each has its own time framework (form a minimum of one year to a maximum of five). These divisions are based on a large analysis of the literature combined with clinical experience. The grandiosity section contains questions about the patient’s functional history and how he or she would describe herself as a person. The main part is composed of specific inquiries that aim to understand whether or not and how the person has unrealistically elevated views of themselves. The interpersonal relations section is largely derived from the psychoanalytic/psychotherapeutic literature. It addresses issues such as the narcissistic person’s tendency to idealize others, to lack empathy, to feel entitled, and to have an exploitative style. The reactiveness section investigates whether a person has exaggerated reactions to criticism, defeat, or disappointment. Specific inquiries try to understand if such reactions are motivated by envy, and whether they result in feeling shame, humiliation, and rage. The affects and mood states section assesses the presence of sustained and deep feelings of emptiness, boredom, meaninglessness, shame, anger, and futility. The section of social and moral adaptation assesses the individual’s capacity to regulate behaviors according to well established moral principles and concern for the others vs. an instrumental, self-serving use of values and morals as well as the and regardless and exploitative attitudes more typically shown by narcissistic individuals.

Other instruments:

The Columbia Suicide Severity Rating Scale (CSSRS) [45] is a scale for the assessment of suicidal ideation in young people aged 12 and over. The scale evaluates the level of suicidal ideation—from thoughts about self-death to the active intention with a plan—and the intensity of suicidal ideation, derived by the subscales of the frequency, duration, controllability, deterrents, and reasons for ideation. The CSSRS also differentiates between the domains of suicidal ideation and those of suicidal behavior, collecting information on the presence and number of suicidal attempts, aborted attempts, and even preparatory acts, and providing indices of the actual and potential lethality of the gesture.

The structured clinical interview for DSM-IV axis II personality disorders (SCID II) [46] is designed to carry out a categorical evaluation of personality disorders in terms of the presence or absence of the disorder, and dimensional evaluation, based on the quantity of diagnostic criterion present or absent. The SCID-II is a clinical interview, consisting of a series of open-ended questions. The clinician is also given the opportunity to use a preliminary questionnaire that facilitates the interview by highlighting the most relevant areas to be investigated.

The Kiddies Schedule for Affective Disorders and Schizophrenia for School-Age Children–Present and Lifetime Version (KIDDIE–SADS–PL) [47] is a diagnostic interview for the evaluation of psychopathological disorders (past and present) in children and adolescents according to the DSM-IV criteria. It is a clinician report interview administered to both children and their parents that allows for an overall score that takes into account all the data collected from the various sources available (family members, children, teachers, pediatricians, etc.). The K-SADS-PL consists of several parts: (I) Unstructured introductory interview; (II) Diagnostic screening interview; (III) Diagnostic Supplement Administration Checklist (IV) Five Diagnostic Supplements (for mood disorders, psychotic disorders, anxiety disorders, attention deficit, and disruptive behavior disorders, substance abuse); (V) Overall checklist of the patient’s medical history; (VI) Scale for the global assessment of the child’s current functioning.

### 2.2. Sample Recruitment

A sample of 103 adolescents aged between 12 and 18 was recruited with the method of subsequent admission between 2018 and 2020 at in-patient and Day Hospital Units of Italian Adolescent Mental Health Services. All patients included in the sample had been referred to one of the two services either for a suicide attempt or for suicidal risk (previous suicidal ideation accompanying other possible mood or behavioral symptoms, impulsive behavior, self-harm, etc.).

Only patient scoring over a cut-off of 2 at the CSSRS (=2 suicidal ideation) were included in the sample.

All patients that met diagnostic criteria for any psychotic disorders group as assessed by DSM-V (e.g., schizophrenic spectrum disorder, schizo-affective disorders, etc. [48], or scoring below 90 on IQ measures were excluded.

At T1, all patients were administered with CSSRS, Kiddie SADS, SCID II, DIN (receiving both a categorical and dimensional diagnosis, which is, number of criteria).

The suicidal risk assessment obtained through the CSSRS retrospectively investigates the appearance of suicidal ideation or conducts and has to be held valid for three months preceding the session of administration.

As far as the suicidal conducts are concerned, a six-month follow-up was carried out evaluating the appearance of a first suicide or further suicide attempt. Among the patients included in the sample, the suicidal conducts variables included in the database refer to the subject’s overall course of suicidal gestures at T2 (from three months before the intake to six months after T1 assessment): the suicidal conduct variables are then the ones finally evaluated at T2.

Hence, the overall clinical sample of 103 adolescents was divided into two clinical subgroups: (a) Stay-ideators: subjects reporting suicidal ideation (above 2 on CSSRS) at T1 and having not attempted suicide from three months before to six months after the first assessment (at T2 they receive 0 for the suicidal conduct variables); (b) attempters: subjects having committed at least one suicide attempt from T1 to T2.

### 2.3. Analysis

SPSS (IBM, Armonk, NY, USA) was used for the statistical analysis. First, a preliminary bivariate correlational analysis was run to understand the association between the suicidality dimension (CSSRS) and narcissistic dimension (DIN). Then, a *t*-test analysis for independent samples was run to understand which narcissistic dimension differentiates the ideators sub-group from the attempters sub-group. A second *t*-test analysis for independent samples was run to understand which narcissistic dimension differentiates the attempters who made a high lethality attempt from those who had made a low lethality attempt. A linear regression model was run to verify which narcissistic dimension contributes to explain the variance of the suicidal behavior indexes. Only the variables found to be significant in the previous analyzes were included in the model. The presence of a mood unipolar or bipolar disorder was included in the model since they are typically indicated in the literature as prominent risk factors. The presence of narcissistic personality disorder was also included, in order to consider its effect combined with the DIN variables.

## 3. Results

Descriptive analyses are shown in Table 1.

### 3.1. Association between Narcissism and Suicidiality

A preliminary bivariate correlational analysis was run. The results are shown in Table 2. The DIN subscale Grandiosity is significantly associated with potential lethality r = 0.215; *p* = 0.030. The DIN subscale affects and mood is significantly associated with potential lethality r = 0.211; *p* = 0.032 and with both severity and intensity of the suicidal ideation (r = 0.241; *p* = 0.014; r = 0.333; *p* = 0.001).

A *t*-test analysis for independent samples was run to understand which narcissistic dimensions differentiate the ideators group from the attempters group. Grandiosity significantly differentiates the ideators group from the attempters group t(101) = −2.361, *p* = 0.020, where attempters (M = 4.51; SD = 2.867) seem to have higher grandiosity scores than ideators (M = 3.20, SD = 2.729). Moreover, affects and mood significantly differentiates the ideators group from the attempters group t(101) = −2.046, *p* = 0.043, where attempters (M = 5.07; SD = 2.725) seem to have higher affects and mood scores than the ideators (M = 4.00; SD = 2.530).

A *t*-test analysis for independent samples was run to understand which narcissistic dimensions differentiate the attempters who made a high lethality attempt from those who had made a low lethality attempt. Only Grandiosity significantly differentiates the low lethality attempters group (M = 3.79; SD = 2.672) from the high lethality attempters group (M = 5.68; SD = 3.045) t(48) = −2.342, *p* = 0.023, where the higher the attempt’s lethality score, the higher the grandiosity scores.

### 3.2. Regression Model

A linear stepwise regression model was run to verify which narcissistic dimension contributes to explain the variance of the suicidal behavior indexes. The results are shown in Table 3. The independent variables chosen are Grandiosity and Affects and mood since they were significant in the previous analyses; the presence of a mood unipolar or bipolar disorder because they are typically indicated in the literature, and the presence of narcissistic personality disorder, to consider its effect. The models tested with suicide attempt and number of suicide attempts as dependent variables were not significant. Grandiosity is the only included significant predictor of potential lethality in the model R^2^ = 0.054; *p* = 0.02; β = 0.233, *p* = 0.02.

## 4. Discussion

As indicated in the introduction, the literature in adult suicidal individuals has evidenced a link between pathological narcissism and suicidality, but some questions still need clarification. This study aimed at investigating the contribution of pathological narcissism and its dimensions to the subsequent stages of the suicidal process in a sample of adolescents through three specific research objectives. With reference to these three objectives, the results seem to confirm what was already shown in the studies on adults and to add some useful empirical information to some of the research questions raised in the literature.

Firstly, the association between the diverse clinical areas of pathological narcissism and the severity and intensity of suicidal ideation has been evaluated. The results show that the increase in the narcissistic affects and mood (e.g., shame, rage, envy, emptiness, boredom) is significantly associated with an increase in the intensity and severity of suicidal ideation. This result is in tally with the clinical view positing a specific function of suicidal ideation for the narcissistic individuals’ emotional regulation [40]. According to this interpretation, narcissistic individuals experience the intensification of specific affects as a condition of internal vulnerability and a specific threat to their self-esteem. Suicidal ideation is then thought to restore a sense of agency and control over emotions, and they become an immediate strategy for recovery of self-esteem [25,49]. As discussed in the introduction, it has been observed that suicidal ideation may preserve some narcissistic individuals from committing actual anti-conservative acts [25]. In keeping with this position, it has been reported that suicidal attempts emerge more sporadically in narcissistic patients and that narcissistic personality disorder cannot be regarded as a strong quantitative predictor of suicidal gestures [37]. Quite similarly, in this study, neither the correlation nor the regression model indicates the narcissistic dimensions as predictors of the emergence of suicidal attempts and the number of suicidal attempts, even though it is possible to highlight, in the bivariate correlations, a tendency towards a positive correlation between grandiosity and suicidal attempt and between affects and mood with suicidal attempt and with the number of suicidal attempts that could reach significance with a larger sample size.

However, other results from this study seem to indicate a more complex relationship between narcissistic pathological functioning and suicidal conduct. In verifying the second and third objectives of this research, results emerging from this sample of adolescents overlap with studies on narcissism and suicidality in the adult population. On a longitudinal level, attempters can be distinguished from stay-ideators subjects because of their significantly higher scores in the dimensions of narcissistic affects and moods and grandiosity. Furthermore, the regression model employing the grandiosity narcissistic dimension has a significant impact on the increase in the lethality of the suicide attempted by the boys and girls of the sample. Within this regression model, it is only the dimension of grandiosity that constitutes a significant predictor of the high lethality of suicidal conduct. The apparent contradiction between the scarce predictive value of NPD for emergence and recurrence of suicidal gestures in general, and the impact of pathological narcissism on the lethality of suicidal attempts has been already highlighted in the literature [37,50]. We contend that the evidence emerging from this study seems to lend further support to an overall perspective concerning the regulative function of suicidality in narcissistic individuals [25,28] and that this perspective can also shed light on the seemingly controversial issues discussed above.

As previously stated, the experience of inner vulnerability due to the increase in narcissistic affects and mood can be initially dealt with by suicidal ideation. The literature indicates that when the state of vulnerability is further increased due to external causes (e.g., life events, interpersonal frustrations, events menacing personal status) or internal factors (e.g., temperamental predisposition or onset of other psychopathological conditions) suicidal ideation may no longer suffice the purpose of self-esteem regulation [25,28,51,52]. The narcissistic individual may thus feel entrapped in a state of helplessness, personal annihilation, confusion between self-hatred (narcissistic rage), and rage toward meaningful others [7,53,54,55]. In keeping with the results from this study, clinical approaches suggest that compensatory grandiose fantasies may constitute the only way to achieve a new sense of mastery and control in this state of entrapment [28] In particular, in severely pathological narcissistic functioning, the defenses of omnipotence, denial and projective identification are the only strategies available to rid the unbearable feelings of shame, weakness and exposure through the suicidal gesture [54,56].

The investigation of suicidal ideation and motivation in narcissistic individuals explicitly includes contents such as fantasies of escape from unbearable frustrations, the denial of real or imaginary conditions of physical or psychic inferiority, supremacy over bodily needs and emotions, the assertion of superiority and self-sufficiency over affective bonds, and vulnerability to losses and separations. Additional contents are the return to a state of perfection and self-ideal, the revenge and triumph over malignant others, to demonstrate the strength to overcome the fear of death, and to survive one’s physical disappearance [27,49,57]. It is important to note, that in our sample of adolescents, those who commit suicide can be distinguished from those who do not by a parallel increase in narcissistic affects and grandiosity. Therefore, the coexistence of intense feelings of vulnerability and the ensuing grandiose compensation can precipitate the suicidal gesture. Furthermore, as clearly shown in this sample, the increase in the grandiose fantasies is accompanied by a detachment from feelings of fear which weakens the realistic evaluation of the consequences of the suicidal gesture and contributes to increasing the lethality of the suicidal attempt [28]. This condition of internal splitting is thought to be responsible for the dissociative state and emotional detachment that may explain the features of narcissistic suicidal attempts: the apparent immediacy and absence of previous signals, its careful, “cold “and “rational” planning of its often lethal outcome [25]. It thus seems that the typical narcissistic modes of self-esteem regulation can account for the differences observed in the more recurrent but less lethal suicide attempts exhibited by borderline patients [16,58]. Borderline patients report suicidal motivations that are more oriented to help-seeking and control over relationships as well as by a general background of emotional instability, less structured defenses, mainly cantered on impulsive acting out [54,59].

Some limits to this study should also be taken into consideration that may also represent relevant guidance for further research. In particular, the recruited sample did not include subjects who finally succeeded in committing suicide. Secondly, the window of longitudinal observation is definitely too short (nine months—considering the three months preceding the time of administration of the instruments and the following six months of the second observation) to leap to generalizable conclusions as to the predictive value of our observations. Thirdly, analysis by gender, performing the correlations between women and men separately, could yield interesting and different results. Finally, a comparison with other personality pathological conditions could have been included, also considering the level of comorbidity between personality disorders. By the same token, we believe that the dimensional assessment of pathological narcissism deserves a secluded study. Since external frustrations and life events have a role in triggering the narcissistic affective dysregulation and possible ensuing recourse to suicidality [26], the next step of research should evaluate the interaction between specific external conditions, namely, past and recent trauma, academic and sentimental failures, losses, and the pathological functioning we have scrutinized in this research.

Furthermore, the considerations concerning the role of pathological narcissism in shaping the suicidal ideational process could be analyzed in more detail. In particular, the link between the dimensions of the DIN and the motivational profiles for suicide identified by our research group can be empirically investigated in a future study [58].

## 5. Conclusions

The understanding and management of suicidal risk in adolescence is a relevant challenge for any mental health professional. Consistent literature has pointed to the role of personality pathology to increase suicidal risk in both adulthood and adolescence [17,59]. In this study, some relevant evidence has been produced that indicates which specific characteristics of narcissistic pathological functioning contribute to the deployment of the suicidal process in this phase of life, replicating and specifying indications coming from clinical and empirical literature in adulthood. Suicidal attempts are reported to be facilitated by specific precipitating events as well as temperamental, psychopathological, and contextual issues. The self-regulatory model of suicidality presented in this paper can serve as an important framework to understand and monitor the impact of other risk factors for suicidality on narcissistic individuals.

In particular, the level of narcissistic vulnerability emerging through specific affects and mood should be taken into account in the first place. The perception of an affective dysregulation engendered by internal events or external conditions seems to be the starting point of the volitional stage of the suicidal process in adolescents with pathological narcissism. The progressive intensification of affective dysregulation along with a concurrent increase in grandiose compensation represents an aspect to be carefully assessed when gauging the likelihood of a passage to actual suicidal attempts. Clinicians should also pay attention to the level of splitting between the intensification of the perception of narcissistic affects and grandiosity and the ensuing level of dissociation. This perspective of evaluation can provide a useful integration for the assessment, which can be more naturally embedded in the daily practice of clinicians involved in the challenging task of managing the clinical risk of a highly unpredictable population such as that one of adolescents.

Finally, we believe that this approach may prove particularly effective in detecting the impact that the current conditions, engendered by the restrictions and life events due to the pandemic, may have on the potentially altered narcissistic functioning inherent to adolescent development [60].

## Figures and Tables

**Table 1:** Descriptive analysis of the sample.

	Frequency	Percentage	Mean	S.D.
Age			15.53	1.123
Female	78	75.7	-	-
Male	25	24.3	-	-
Ideators	46	44.7	-	-
Attempters ^4^	67	55.3	-	-
N suicide attempts			0.79	1.006
Attempters low lethality ^1^	28	27.2	-	-
Attempters high lethality ^1^	22	21.4	-	-
Mood disorders ^2^	86	84.3	-	-
Bipolar disorders ^2^	13	12.7	-	-
Anxiety disorders ^2^	37	36.3	-	-
Substance abuse ^2^	24	23.5	-	-
Grandiosity ^3^	-	-	3.92	2.869
Interpers. Relations ^3^	-	-	5.98	2.657
Reactiveness ^3^	-	-	5.21	2.163
Affects and mood ^3^	-	-	4.59	2.680
Moral adaptation ^3^	-	-	1.27	1.610

^1^ We divided the sample according to the CSSRS index of potential lethality of suicide attempts. A low lethality attempt is not likely to end in serious injury or death; a high lethality attempt is likely to end in major injury or death. ^2^ Presence/Absence of the disorder according to KIDDIE-SADS-PL. ^3^ DIN scale row score. ^4^ Two subjects committed a suicide attempt during the six months follow-up.

**Table 2:** Correlational analysis grandiosity, interpersonal relations, reactiveness, affects and mood, moral adaptation, with CSSRS suicidality scales.

	Ideation Severity	Ideation Intensity	Suicidal Attempt	Number Suicidal Attempts	Potential Lethality
Grandiosity	−0.068	−0.153	0.171	0.096	0.231 *
Interpers. Relations	0.056	0.031	−0.022	0.006	0.125
Reactiveness	0.004	0.050	0.006	0.039	0.006
Affects and mood	0.241 *	0.333 **	0.143	0.182	0.211 *
Moral adaptation	−0.069	−0.126	−0.005	0.012	0.062

* *p* < 0.05, ** *p* < 0.01.

**Table 3:** Regression analysis.

Dependent: Potential Lethality								
	B	SE	β	*p*	R^2^	R^2^ Change	F	*p*
Total model					0.054	0.054	5.568	0.02
Grandiosity	0.070	0.143	0.233	0.020				
Excluded variables								
Affects and mood	0.093			0.351				
Mood disorders	0.156			0.120				
Bipolar disorders	0.187			0.059				
NPD	−0.059			0.587				

N = 99; SE = standard error of B. Mood disorders = diagnosis of any unipolar mood disorder. Bipolar disorders = diagnosis of any bipolar disorder. NPD = diagnosis of narcissistic personality disorder.

## Data Availability

The data have not so far been made available on any shared platform, because of the sensitivity of the matter under investigation. If the Editors or the Reviewers will require the consultation of the database, we are ready to send it in an anonymous form and taking into account the aspects of the sensitive pieces of information.
